# Minimally Invasive Facial Lifting Using the Versatility of Calcium Hydroxyapatite (STIIM)

**DOI:** 10.1111/jocd.70427

**Published:** 2025-09-09

**Authors:** Barbara Saavedra Deus, Renata Viana

**Affiliations:** ^1^ Independent Researcher São Paulo Brazil

**Keywords:** calcium hydroxyapatite, facial lift, skin laxity

## Abstract

**Introduction:**

Facial aging is a multifactorial process characterized by skin laxity, volume loss, and collagen degradation. Calcium Hydroxyapatite (CaHA) is a versatile biostimulatory filler that can provide both structural support and collagen stimulation. This study evaluates a novel technique using CaHA with tailored dilutions for minimally invasive facial rejuvenation, focusing on key ligamentous structures.

**Objectives:**

To assess the aesthetic outcomes, patient satisfaction, and safety profile of the technique using regionalized CaHA dilutions.

**Methods:**

A prospective, single‐center study was conducted involving 20 subjects with facial laxity. CaHA injections were performed using three distinct dilution ratios protocols, targeting the zygomatic, temporal, and mandibular regions. Endpoints were evaluated through Face‐Q questionnaires, GAIS, and standardized 2D and 3D imaging.

**Results:**

At 90 days, 94% of subjects reported aesthetic improvement, and the mean Face‐Q Skin score increased from 43.2 ± 14.7 to 74.9 ± 14.7. These results were maintained at 360 days, with 94% of participants continuing to report improvement and a sustained FACE‐Q Skin score of 77.2 ± 16.4. Investigator assessments confirmed long‐term improvements, and 3D imaging showed persistent enhancement in facial contours. Adverse events were mild and self‐limiting.

**Conclusions:**

This CaHA‐based technique offers a safe, minimally invasive facial lifting effect with sustained improvement over 12 months. Its effectiveness is enhanced by targeting facial ligaments and customizing dilution based on anatomical region.

## 1 Introduction

Facial aging results from the progressive degradation of collagen types I and III, leading to skin laxity, volume loss, and wrinkle formation [1]. A hallmark of this process is the decline in collagen synthesis, which contributes to the thinning and weakening of the skin's extracellular matrix, accelerating the appearance of aging. Collagen biostimulators have emerged as promising agents for skin regeneration and aesthetic enhancement [2]. Among them, alcium hydroxyapatite (CaHA) is particularly recognized for its ability to promote neocollagenesis, thereby improving skin quality, elasticity, and firmness [3].

The current trend in regenerative medicine reflects a growing preference for minimally invasive techniques that stimulate the body's own resources for tissue reconstruction [4]. This approach aligns with the demand for nonsurgical facial rejuvenation solutions that offer aesthetic benefits without the risks and recovery time associated with surgical interventions [5, 6, 7]. CaHA has garnered attention for its dual role enhancing both tissue support and, when hyperdiluted, acting as a collagen stimulator in facial and non‐facial areas [4].

This study introduces a minimally invasive technique that applies CaHA in three distinct dilution ratios targeting facial ligaments to provide tissue structural support and lifting while respecting anatomical safety zones.

## 2 Materials and Methods

### 2.1 Study Design and Ethical Considerations

A prospective, single‐center study approved by the local ethics committee (approval no. 6.613.054), was conducted in accordance with the Declaration of Helsinki. All subjects provided informed consent prior to enrollment. Participants were monitored through satisfaction questionnaires, clinical evaluations, standardized photographs, and adverse events recordings.

### 2.2 Study Population

Twenty adults (aged 34 to 54 years old) with facial laxity were included. Subjects with prior facial filler use, biostimulating invasive procedures within 12 months prior to the procedure, active dermatological conditions, or immunosuppression were not included. Participants were also committed to refraining from other facial procedures during the course of the study.

### 2.3 Product Details

The product used was STIIM (by Ilikia, manufactured by CGBIO, South Korea). It is composed of 30% CaHA microspheres in carboxymethylcellulose gel (70%), available in 1.5 mL prefilled syringes.

### 2.4 Procedure

#### 2.4.1 Pre‐Treatment Preparation

The treatment areas were marked, the skin cleansed, and local anesthesia with 2% lidocaine was applied at the entry points.

#### 2.4.2 Dilutions and Injection Technique

**FIGURE 1:**
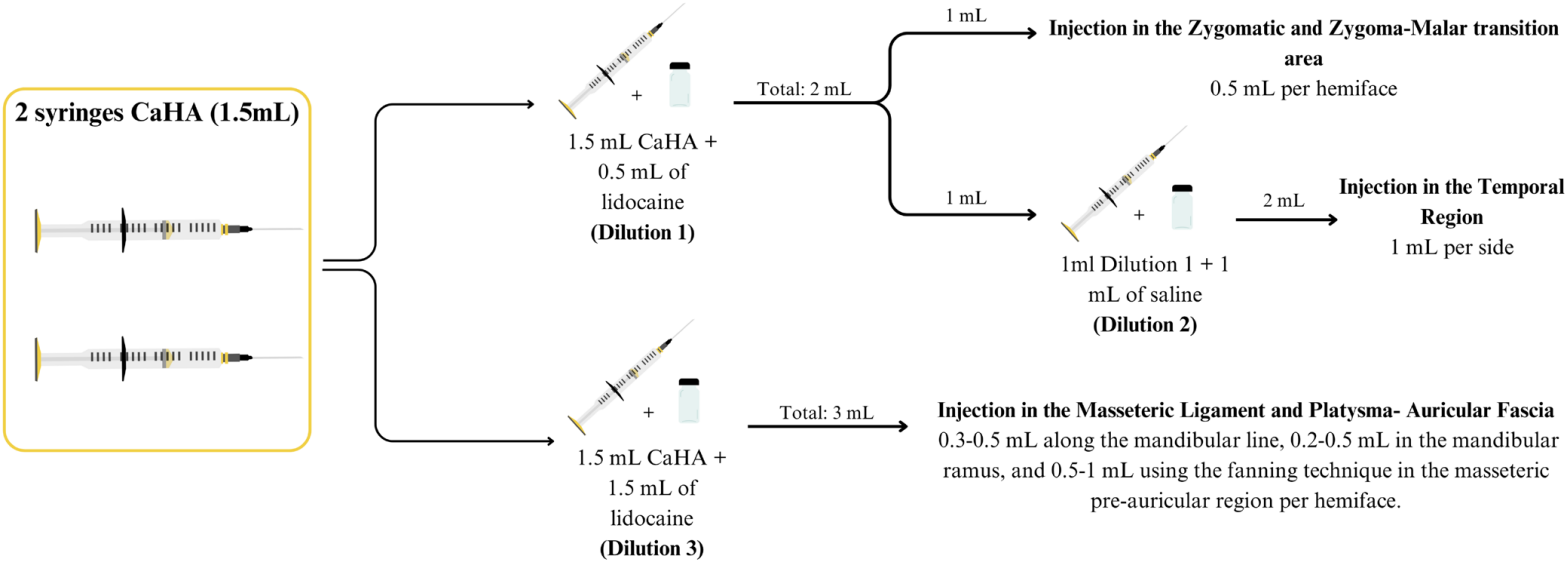
Schematic representation of the CaHA injection protocol. Diagram of entry points and CaHA dilutions: Dilution 1 (1.5 mL CaHA +0.5 mL lidocaine) for the zygomatic area; Dilution 2 (Dilution 1 + 1 mL saline) for the temporal area; Dilution 3 (1.5 mL CaHA +1.5 mL lidocaine) for the mandibular contour. Each injection area and volume is indicated per hemiface.

All procedures were performed using a 22G × 50 mm blunt‐tip cannula to improve safety, and the CaHA was applied with varying dilutions, leveraging the product's versatility based on target facial anatomical areas (see Figure 1).

**FIGURE 2:**
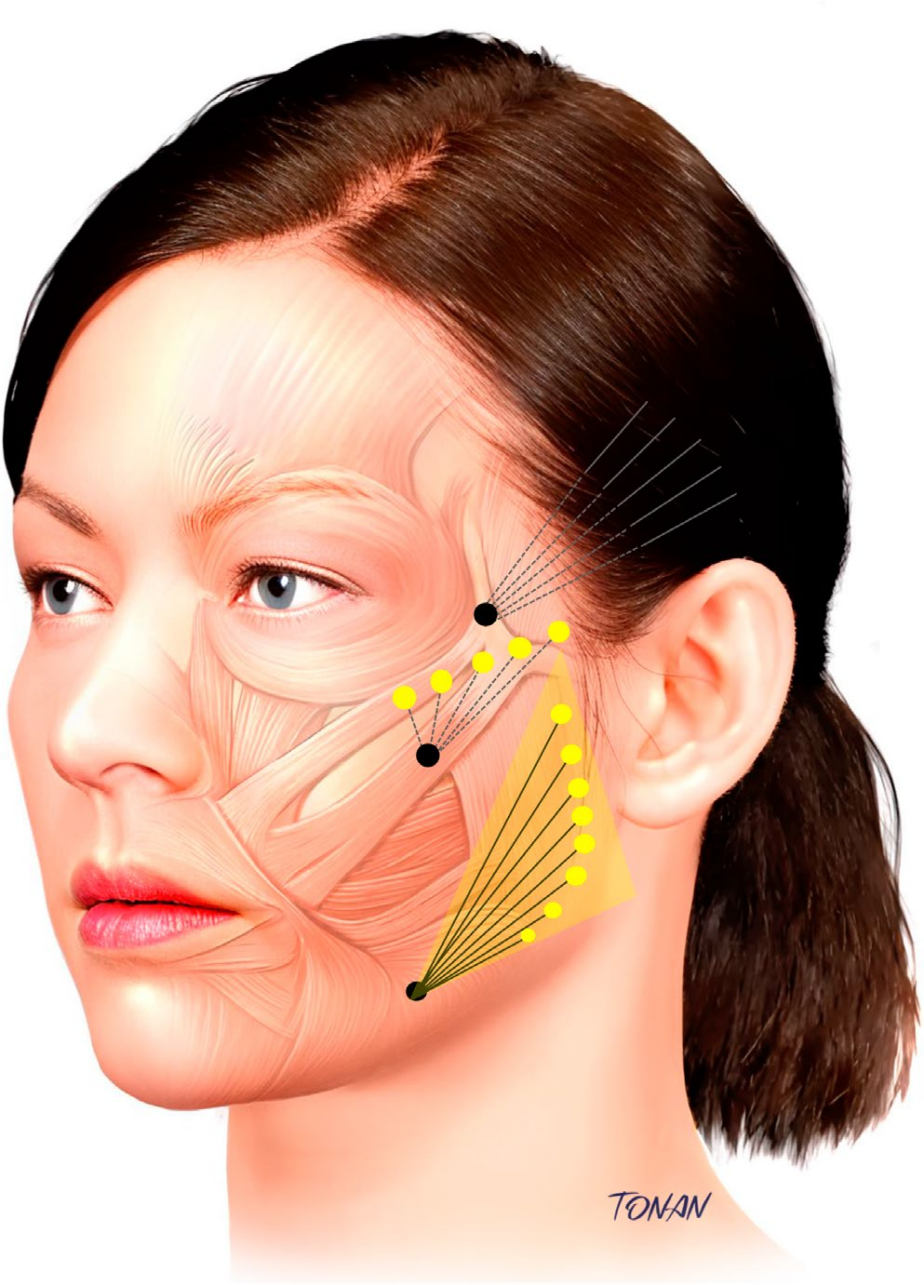
CaHA injection technique. (i) Temporal region: Cannula entry point (black dots) in the anterior zygomatic area, with retroinjection (solid lines) performed above the superficial temporal fascia. (ii) Zygomatic and zygoma‐malar transition area: Supraperiosteal microbolus injections (yellow dots). (iii) Mandibular and platysma‐auricular fascia area: Retroinjections in the mandibular contour and in the platysma‐auricular fascia area. Medical illustration by Rodrigo Tonan.

Procedure guidance markings are illustrated in Figure 2, which outlines entry points and CaHA dilution strategies: Dilution 1 (1.5 mL CaHA +0.5 mL lidocaine) for the zygomatic region, Dilution 2 (further diluted with saline) for the temporal area, and Dilution 3 (1.5 mL CaHA +1.5 mL lidocaine) for mandibular contour.

##### 2.4.2.1 Zygomatic and Zygoma‐Malar Region

Zygomatic area and zygoma‐malar transition region are addressed in this first step. The main purpose of this step is to provide support with immediate lifting effects. A volume of 0.5 mL per side of Dilution 1 (1.5 mL of CaHA mixed with 0.5 mL of 2% lidocaine) was used in the supraperiosteal zygomatic plane, applying microbolus injections.

##### 2.4.2.2 Temporal Region

Posterior superficial temporal fascia is addressed in order to enhance the lifting effect based on the principle of redensification of the treated facial ligaments [5, 8]. For safety, due to the increased risk of injury, a more diluted product was used, and an entry point was set at the anterior zygomatic area, perpendicular to the superficial temporal artery [9]. Thus, the residual 1 mL of diluted CaHA from Dilution 1 is further diluted with 1 mL of saline to obtain 2 mL of re‐diluted product (Dilution 2; 1:67 dilution rate), which is injected retrogradely above the superficial temporal fascia using a fan‐shaped distribution pattern, applying 1 mL per side.

##### 2.4.2.3 Mandibular Contour and Platysma‐Auricular Fascia Region

To enhance mandibular contour and stimulate collagen production in the masseteric and platysma‐auricular fascia region, a second syringe of CaHA (1.5 mL) is diluted with 1.5 mL of lidocaine (Dilution 3, see Figure 2), resulting in a 1:1 dilution ratio. The entry point was located at the post‐jowl area on the jawline. A retroinjection of 0.3–0.5 mL per side was delivered along the mandibular line on each side, followed by an additional 0.2–0.5 mL injected along the ramus and 0.5–1 mL retroinjected in the masseteric and platysma‐auricular fascia region.

### 2.5 Assessment Tools

This study employed assessment scales to evaluate clinical outcomes and patient satisfaction following CaHA injections. The following assessment scales were used:

#### 2.5.1 Global Aesthetic Improvement Scale (GAIS)

Overall facial aesthetic improvement was assessed through GAIS from the investigator's perspective. This scale rates the improvement as “worse,” “no change,” or “improved”, “much improved” and “very much improved” [10].

#### 2.5.2 Patient Satisfaction Scales

The Face‐Q scales (Skin and Outcome domains) were used to assess patient‐reported satisfaction regarding skin quality and overall satisfaction with the aesthetic results [11]. These assessments were conducted at baseline and follow‐up visits to capture subjective improvements from the participants' perspective. Also, patient satisfaction was assessed with a 5‐point Likert scale at baseline (D0) and at 90‐ and 360‐day follow‐up.

#### 2.5.3 Photographic Documentation

Standardized clinical photographs using 2D and 3D images (obtained with the Quantificare system) were taken before the procedure and during follow‐up visits.

#### 2.5.4 Adverse Events Monitoring

Throughout the study, adverse events (AEs) were recorded and categorized based on severity, duration, and potential relation to the device or procedure.

## 3 Results

### 3.1 Participants Demographics

A total of 20 participants (mean age of 45.45 ± 6.39; range 34–54 years) were enrolled, 14 (70%) of whom were female, and 5 of the women were postmenopausal. The mean Body Mass Index (BMI) was 27.16 ± 5.25 kg/m^2^. Participants had no prior facial interventions in the preceding 12 months (Table 1).

**TABLE 1:** Baseline demographic and clinical characteristics of subjects (*n* = 20).

Participants demographic data (*N* = 20)	
Variable	*n* (%) or Mean ± SD (range)
**Age (years)**	45.45 ± 6.39 (34–54)
**Gender**	
Female	14 (70%)
Male	6 (30%)
**Fitzpatrick scale**	
Type I	1 (5%)
Type II	3 (15%)
Type III	7 (35%)
Type IV	6 (30%)
Type V	2 (10%)
Type VI	1 (5%)
**BMI (kg/m** ^**2**^ **)**	27.16 ± 5.25 (19.16–39.67)
**Sun exposure**	
Mild	8 (40%)
Moderate	12 (60%)
**Daily Facial Sun protection**	10 (50%)
**Menopause**	5 (35.7%)
**Smoking**	2 (10%)
**Facial laxity rating scale**	
*Mild*	
Class 1	0
Class 2	6 (30%)
Class 3	6 (30%)
Class 4	6 (30%)
*Moderate*	
Class 5	1 (5%)
Class 6	1 (5%)
*Severe*	
Class 7–9	0

### 3.2 Patient‐Reported Outcomes

**FIGURE 3:**
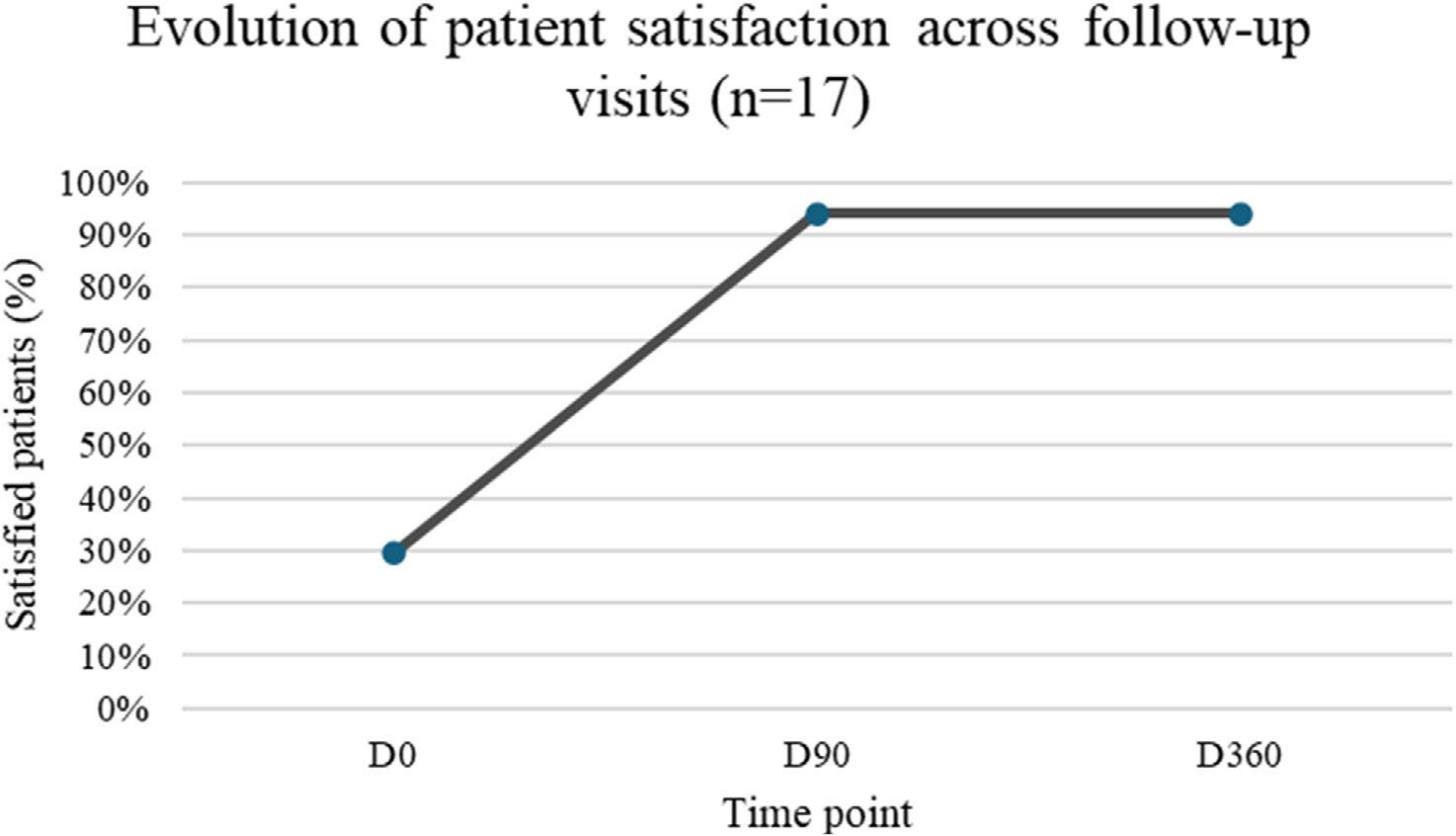
Evolution of patient satisfaction over time (*n* = 17). Percentage of patients satisfied with facial appearance at baseline (D0), 90 days (D90), and 360 days (D360). Satisfaction increased from 29% at baseline to 94% at D90 and was maintained at D360.

Patient satisfaction was assessed at baseline and at each follow‐up visit using a 5‐point Likert scale. Satisfaction rates increased from 29% at baseline (D0) to 94% at 90 days (D90), and the improvement was mantained at the 360‐day follow‐up (D360), as shown in Figure 3. Three participants were excluded from the analysis due to significant weight fluctuations (weight gain > 10% of body weight) or loss to follow‐up, resulting in a final sample of 17 for outcome evaluation.

**FIGURE 4:**
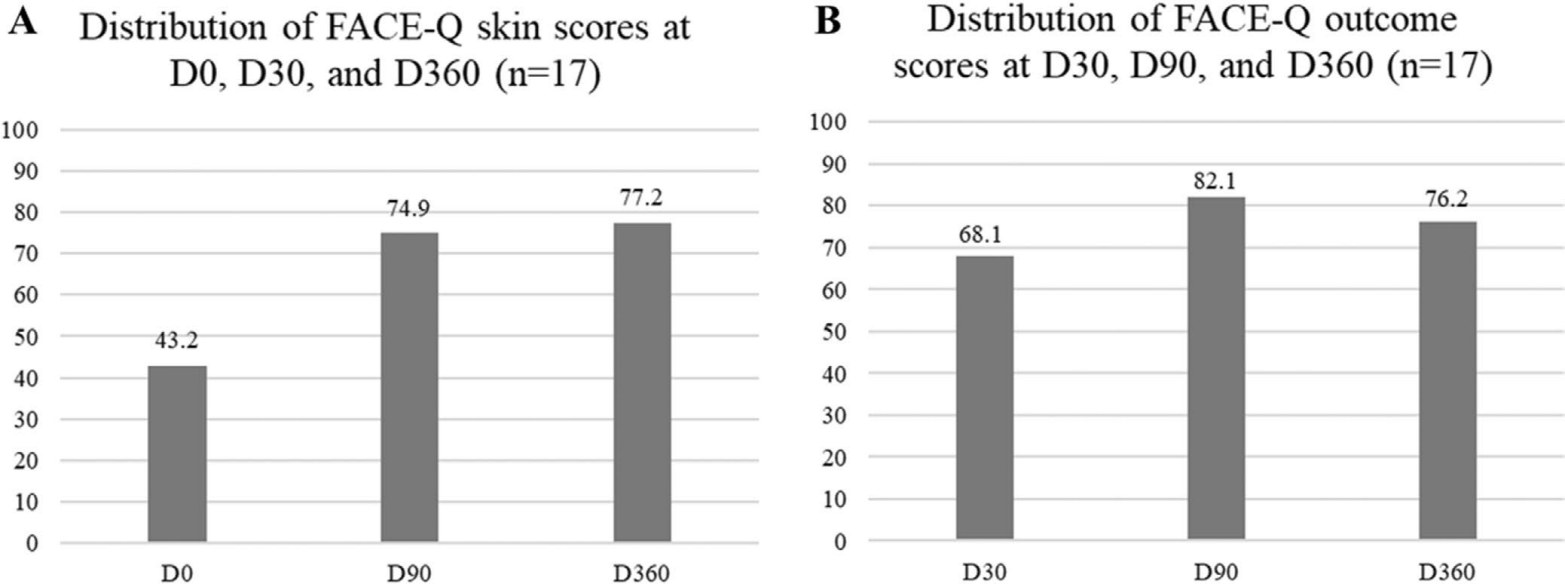
Face‐Q assessment scores at different timepoints. (A) Skin domain score improved from 43.2 ± 14.7 (D0) to 74.9 ± 14.7 (D90) and 77.2 ± 16.4 (D360). (B) Outcome domain score rose from 68.1 ± 31.6 (D30) to 82.1 ± 17.0 (D90) and remained at 76.2 ± 18.6 (D360).

Face‐Q Skin Scores improved significantly from 43.2 ± 14.7 (D0) to 74.9 ± 14.7 (D90) and further sustained at 77.2 ± 16.4 (D360) (Figure 4A). Face‐Q Outcome scores also increased from 68.1 ± 31.6 at D30 to 82.1 ± 17.0 at D90, and were maintained at 76.2 ± 18.6 at D360, demonstrating long‐term stability of results (Figure 4B).

### 3.3 Investigator‐Assessed Outcomes

GAIS evaluations by three investigators confirmed the findings. At the 90‐day follow‐up (D90), standardized photographs of 17 participants were assessed, and 76% were rated as showing improvement (6% “very much improved,” 27% “much improved,” 43% “improved”), while at D360, 78% of the nine subjects who returned for photographic evaluation showed aesthetic improvement.

**FIGURE 5:**
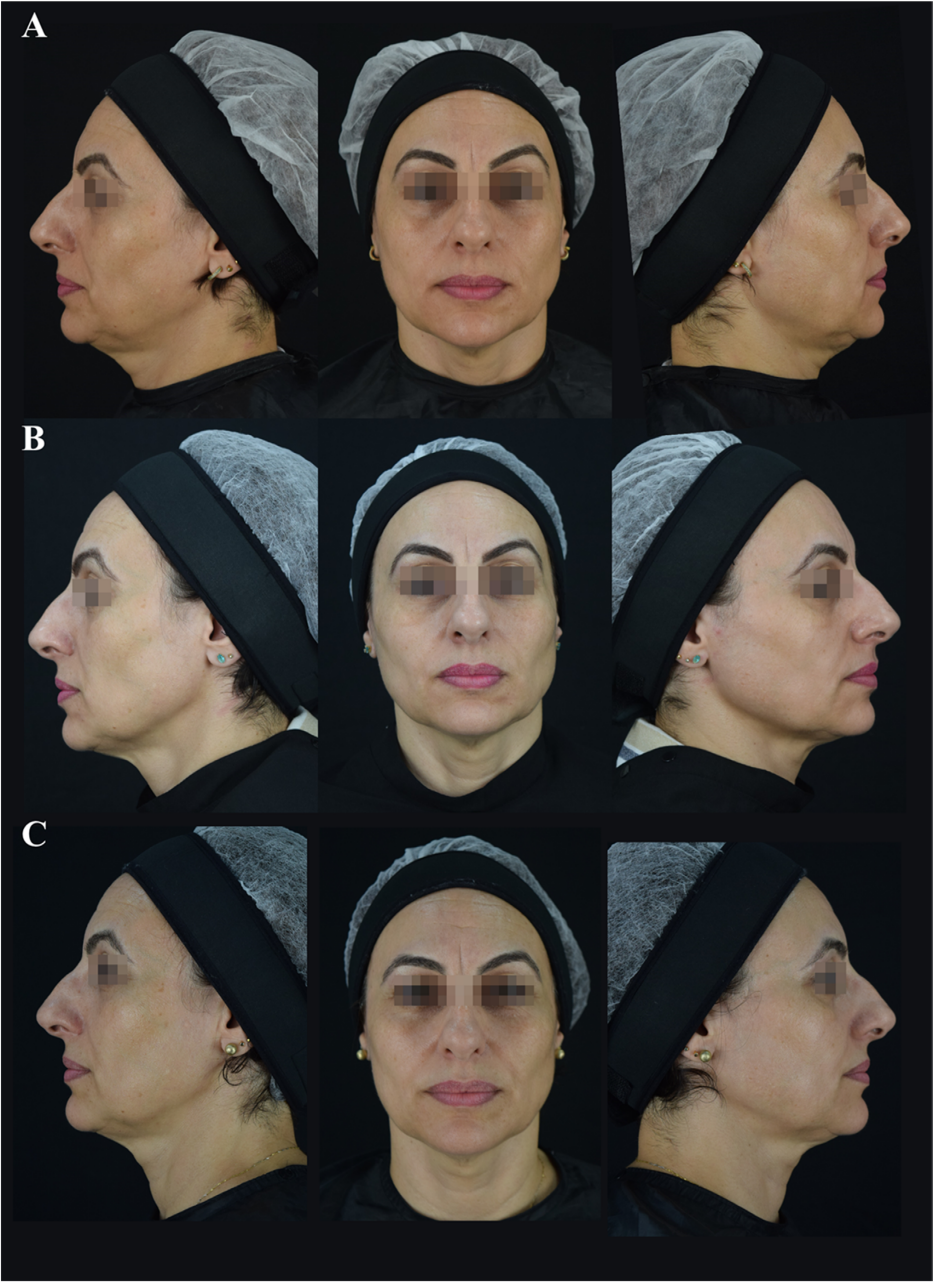
Facial imaging before and after (representative case). Progressive structural improvements observed at baseline (A), 90 days (B), and 360 days post‐treatment (C) using photographic assessment.

**FIGURE 6:**
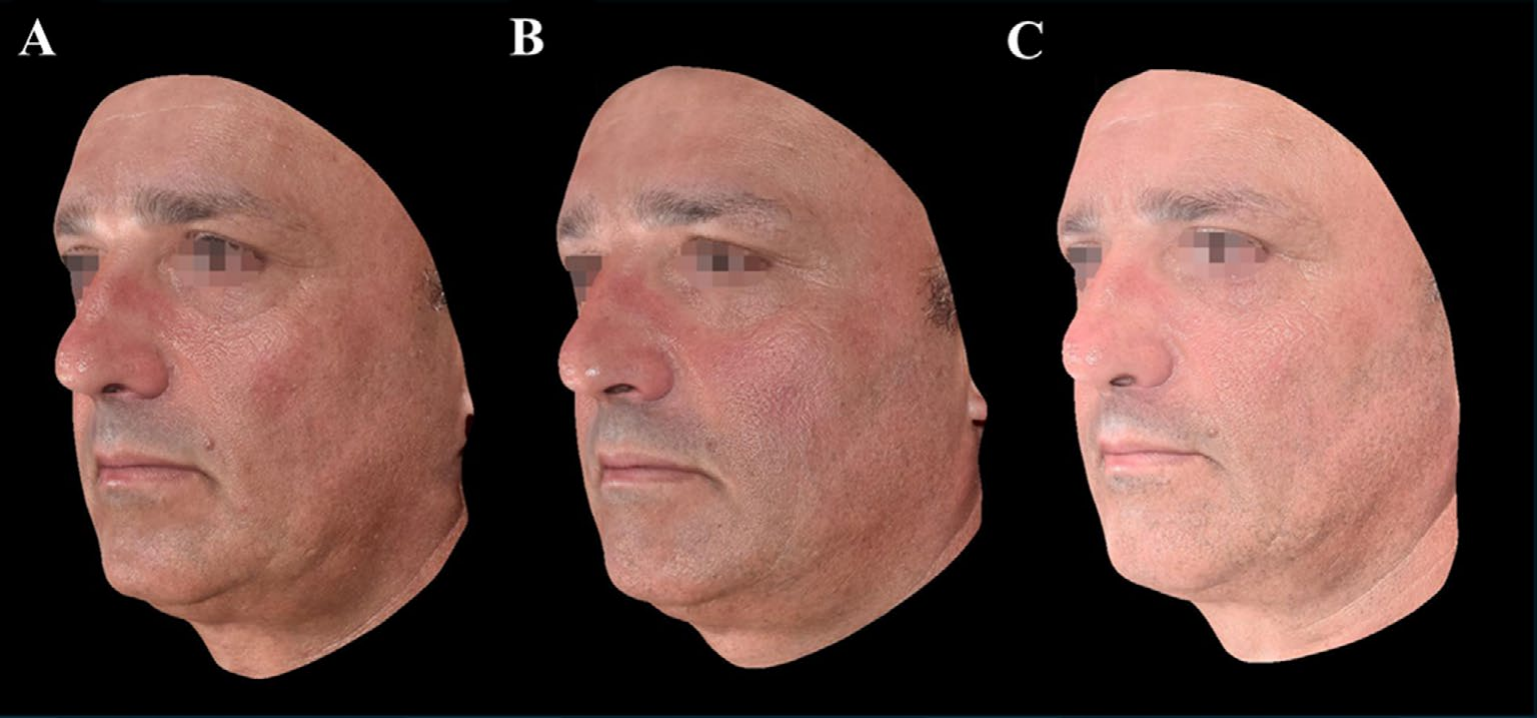
Facial assessment via 3D imaging. Images show pretreatment (A), 90 days (B), and 360 days (C) post‐treatment, demonstrating enhanced jawline contour and reduced nasolabial fold.

Photographic and 3D imaging (Figures 5 and 6) visually supported these findings, demonstrating progressive and sustained aesthetic improvements following treatment with CaHA, with enhanced jawline definition, skin tightening with a lifting effect, and improved facial contours over time.

**FIGURE 7:**
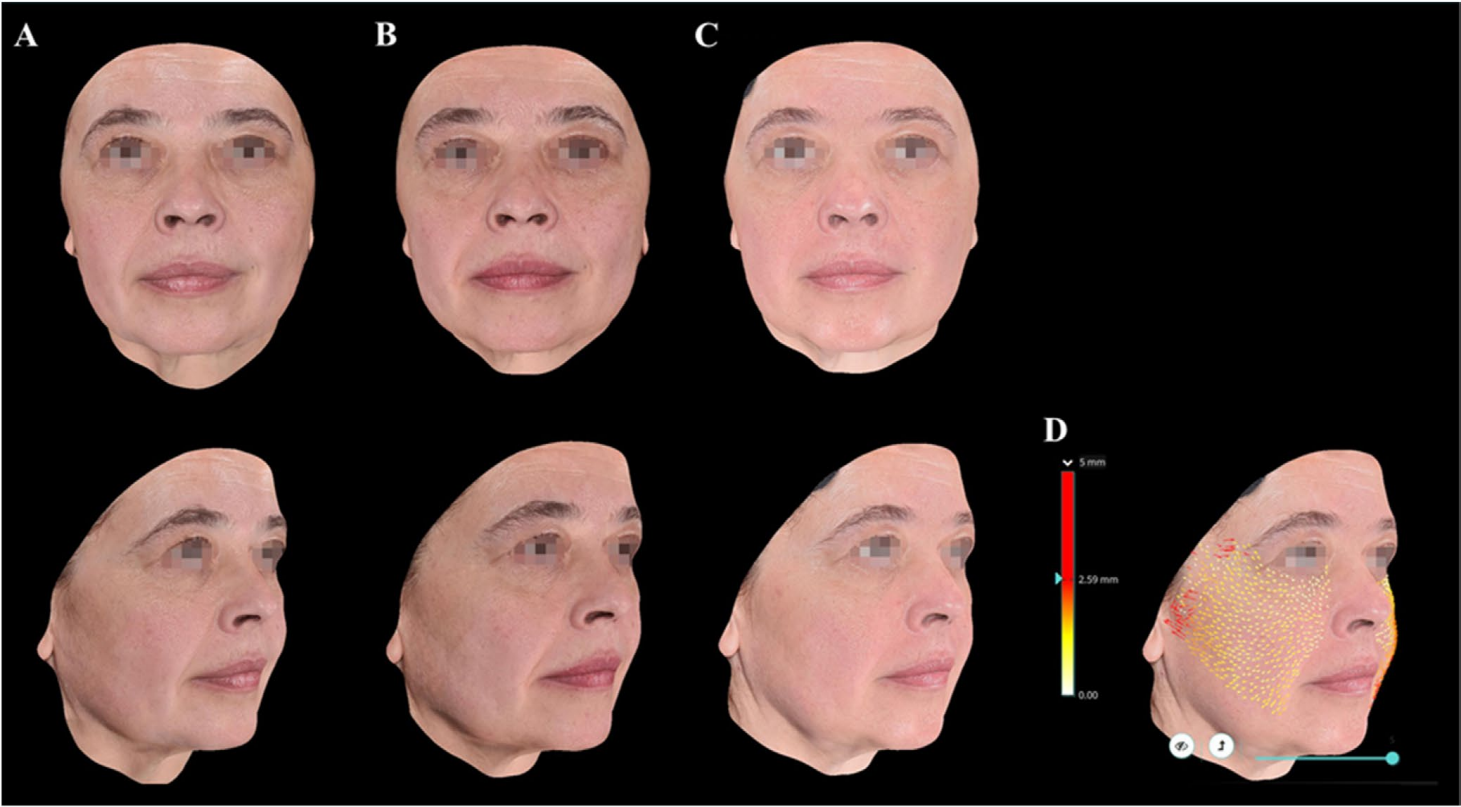
Long‐term lifting effect assessment (360 days). Quantitative visualization with improvements maintained over time. Pretreatment images (A), 90 days post‐treatment (B), 360 days post‐treatment (C), and lifting effect evaluation at 360 days (D).

Three‐dimensional (3D) assessments (Figures 6 and 7) also highlight continuous improvements in skin texture, facial volume, and overall facial contour definition, particularly in the jawline region. Furthermore, a dedicated lifting analysis performed by the software (Figure 7D) demonstrated a sustained lifting effect at the 360‐day follow‐up, with visible improvement in the jawline contour and reduction of the nasolabial fold, confirming the long‐term structural benefits of the treatment.

### 3.4 Monitoring of Adverse Events

The procedure was well tolerated, with no serious adverse events or major discomfort. None of the participants required the use of analgesics or corticosteroids.

One subject reported mild edema within 24 h, and another experienced a palpable transient nodule in the mandibular region (which lasted 1 month and resolved spontaneously), likely related to product accumulation. No late‐onset adverse effects were observed.

## 4 Discussion

This study introduces a minimally invasive technique for facial rejuvenation by employing customized CaHA dilution ratios, targeting key facial ligaments to enhance facial contour and promote long‐term collagen bio‐stimulation. This approach focuses on ligamentous support, consistent with recent anatomical and clinical research publications, which emphasize the role of facial retaining ligaments in minimally invasive lifting procedures [6, 7].

Notably, the use of distinct CaHA dilutions tailored to anatomical zones appears to maximize both immediate lifting and long‐term collagen biostimulation. This strategy aligns with anatomical studies emphasizing the role of facial retaining ligaments in structural support [7, 12, 13]. High satisfaction rates (94%) at 90 days and 12 months, together with improvements on Face‐Q and GAIS, support the clinical utility of this technique.

The 12‐month sustained aesthetic improvements and high patient satisfaction, with no serious adverse events reported, also align with previous studies showing long‐term benefits of CaHA. Green et al. observed meaningful improvements up to 60 weeks post‐treatment, even without retreatment [14], and Bass et al. reported that 40% of patients maintained visible improvements 30 months after injection, with no late complications [15]. These outcomes reinforce the durability and safety of CaHA as a long‐term regenerative treatment.

Among the 20 patients treated, five were postmenopausal. Although a formal subgroup analysis was not performed, these participants subjectively exhibited particularly favorable outcomes, with high satisfaction and pronounced improvements in facial contour, especially in the jawline and midface, as documented in Figures 5 and 7. This observation suggests that postmenopausal skin also exhibits response to collagen‐stimulating treatments. The hormonal changes associated with menopause—such as estrogen deficiency, reduced collagen content, and dermal thinning—are known to significantly alter skin structure and regenerative potential [16]. Further studies are warranted to investigate whether these factors could contribute to differential responses to CaHA in this population.

Moreover, the findings suggest that different dilutions of CaHA play a crucial role in optimizing results. The use of lower dilutions in strategic areas, such as the zygomatic region, enhances immediate lifting effects while maintaining long‐term skin quality improvements. This concept has been supported by rheological and histological studies. McCarthy et al. (2024) demonstrated that CaHA‐CMC undergoes significant rheomodulation with increasing dilution, transitioning from a cohesive, elastic gel suitable for lifting to a fluid‐like material better suited for biostimulation [17]. In parallel, Botsali [18] showed that even highly diluted CaHA (up to 1:3) maintains significant collagen production and fibroblast stimulation, confirming the sustained regenerative potential of diluted formulations. These findings support the rationale for adapting dilution ratios to the anatomical and functional goals of each facial region.

These results are also in agreement with studies demonstrating that temporal injections can contribute to lifting effects, improving midface contour and reducing nasolabial folds and jawline sagging [7, 13, 19]. This study further validates the importance of posterior temporal support in facial lifting procedures, reinforcing the idea that addressing ligamentous anchoring points can lead to broader aesthetic improvements.

A key aspect of our approach is the customization of treatment through tailored dilution ratios aligned with the current consensus that nonsurgical lifting should be adapted to individual anatomical needs, rather than applying a one‐size‐fits‐all filler technique [20]. Throught tailored dilution ratios and inherent versatility of CaHA, combining mechanical lifting and bio‐stimulation, we optimize the results with lifting, and light volumization where necessary, while biostimulating and enhancing tissue support in key ligamentous zones.

Despite promising results, some limitations must be considered. The small sample size limits generalizability. Further studies with larger, controlled, and multicenter cohorts are needed. Histological studies and blinded image scoring could strengthen mechanistic understanding. Additionally, comparison with other lifting modalities (e.g., threads, HA fillers) would clarify the unique benefits of CaHA.

## 5 Conclusion

This study demonstrates that the use of calcium hydroxyapatite (CaHA) in a ligament‐targeted, dilution‐specific injection technique results in effective and sustained facial lifting, with high levels of patient satisfaction maintained over 12 months. These results reinforce the importance of respecting facial retaining ligaments in minimally invasive procedures and suggest that tailored dilution protocols may enhance both structural outcomes and biostimulatory effects. Despite its limitations, the findings support this technique as a promising, long‐term, minimally invasive alternative for facial rejuvenation.

## Conflicts of Interest

Barbara Saavedra is an independent researcher with no conflicts of interest. Renata Viana serves as a scientific consultant for Ilikia. The authors declare no potential conflicts of interest with respect to the research, authorship, or publication of this article.

## Data Availability

The data that support the findings of this study are available from the corresponding author upon reasonable request.
